# Supplementary material for: "Pain in my heart": Understanding perinatal depression among women living with HIV in Malawi
Source: PLoS One. 2020 Jun 5;15(6):e0227935. doi: 10.1371/journal.pone.0227935 (PMC7274419; doi:10.1371/journal.pone.0227935)
Supplement: S1 Data — (DOC) [file pone.0227935.s001.doc]

**Kukhumudwa kobwera pa nthawi yochira ndi pamene mayi ali ndi HIV – Chikalata chotsogolera mafunso a amayi**

**Chidule cha zochitika (cha wofunsa)**: Cholinga cha chikalata cha mafunso ozamamawa ndi chofuna kufufuza zokhudzana ndi maganizo a amayi amene ali ndi HIV wokhudza kukhumudwa kwa amayi amene akuchira (PND), komanso kutolera uthenga wokhudza zisankho zawo pa nkhani younika ndi chithandizo kwa amayi amene ali ndi vuto lokhumudwa pa nthawi imene akuchira (PND).ndi m’mene zasokonezera machitachita awo muchisamaliro cha HIV.Mafunso ozamawa adzaphatikizapo onse amayi amene akuchira ndi amene anachira okhala ndi HIV ndi vuto lakukhumudwa (PND).

-------------------------------------------------------------------------------------------------

**Mau oyamba :**

Muli bwanji, ndikugwira ntchito mmalo mwa wofufuza ku bungwe la UNC -Malawi ndipo tili ndi chidwi chachikulu pa maganizo anu pa ena mwa mavuto anu mu dera lino, makamaka mavuto amene amachita ndi khalidwe la anthu pa nthawi imene ali woyembekezera kapena atangochira kumene mwana):

**Kufotokoza zokhudza kukhumudwa pa nthawi imene mayi akuchira:**

1. Kodi munayamba mwawonapo amayi amene ali ndi vuto ndi zinthu akakhala ndi mimba kapena akangochira kumene zimene anthu ambiri samakhala nazo ndi vuto? Mwachitsanzo, Kuvutika kudya,kuvutika pochita ndi mwana wawo, kapena abwenzi ndi am’mbanja mwawo monga mwa masiku onse (Monga iwo amene amakakhala paokha)? Kuvutika kugwira ntchito za pakhomo kapenanso kumwa mankhwala ozunguza ubongo pofuna kuyiwala mavuto awo? Makamakanso iwo amene akuganiza zodzivulaza okha?
   1. Kodi munthu wotere mungamufotokoze bwanji?

Ngati palibe yankho, fufuzanibe: Kodi mumati chiyani wina akakhala kuti sakusangalala pafupifupi nthawi zonse? Kodi mumatchula kuti chiyani izi zikamachitika panthawi yimene munthu ali woyembekezera?

2..Aliyense amatha kukhala odandaula, kuchulukidwa ndiso wa nkhawa mwa apo ndi apo. Ngakhale zili chomwechi, ngati kudandaula ndi nkhawazo zikuchitika kwa nthawi yayitali kapena kwa muyeso waukulu timanetha kunena kuti ndi matenda okhumudwa/ matenda ankhawa.Mau amenewa munayamba mwawamvapo?

- 1. Ngati ndi choncho, kodi munamva akugwiritsidwa ntchito?
  2. Ngati ayi, kodi inu mungafotokoze bwanji ngati amayi akukumana ndi zokhumudwitsa kapena kuvutika kuti akhale bwinobwino pa nthawi yoyembekezera?

**Zochitika mu nthawi ya kukhumudwa**:

3.Tangoganizirani chochitika kwa mayi wachichepere amene ali ndi vuto la kukhumudwa (depression). Aunikeni ali wosasangalala nthawi ndi nthawi, mwinanso kumangolira pafupipafupi kapenakukhala chete kwambiri kapena kudzichotsa pakati pa anzake akakhala woyembekezera. Tangoganizirani mwana atabadwa, iye kukhala ndi vuto lodzilimbikitsa yekha kusamala mwana , kugwira ntchito za pakhomo, kapena kulandira anzake kapena am’mbanja mwake akabwera kudzawona mwana.

- 1. Kodi munthu wotere amachita naye bwanji ku dera?
  2. Kodi munthu wotere banja lake ndi anzake amachita naye bwanji?
  3. Kodi munthu wotere mwamuna wake amachita naye bwanji?
  4. Mukanakhala kuti inu ndi amene mukukumana ndi mavuto amenewa kodi mukanalankhula ndi ndani za mavutowa?
  5. Mukanakhala kuti inu ndi amene mukukumana ndi mavuto amenewa kodi mukanathana nawo bwanji? Nkhawa kapena zodandaula zin pa nthawi yoyembekezela?
  6. Kodi mukuganiza kuti zina mwa zifukwa zimene mayiyu akukumana nazo zimene zikuyambitsa kukhumudwa (depression)?
  7. Pakanakhala chiyeso chimene chikanapeza munthu chonchi wokhala ndi vuto la kukhumudwa)” kodi mukuganiza kuti angamve bwanji zokhudza zimenezi?

Note: ngati mzimzyi angakambe za HIV pamenepa tengelanipo mwayi ofufuza zambiri za kusiyana komwe kungakhalepo.

4.Tsopano ganizirani chochitika pamene chisamaliro choperekedwa kwa mayi wosachira, kapena pa nthawi imene mwana akubadwa, mayi wadziwa kuti amupeza ndi HIV. Wunikani mayi ali wosasangalala nthawi ndi nthawi, mwinanso kumangolira pafupipafupi kapenakukhala chete kwambiri kapena kudzichotsa pakati pa anzake akakhala woyembekezera. Tangoganizirani mwana atabadwa, iye kukhala ndi vuto lodzilimbikitsa yekha kusamala mwana , kugwira ntchito za pakhomo, kapena kulandira anzake kapena am’mbanja mwake akabwera kudzawona mwana.

- 1. Kodi munthu wotere amachita naye bwanji ku dera?
  2. Kodi munthu wotere banja lake ndi anzake amachita naye bwanji?
  3. Kodi munthu wotere mwamuna wake amachita naye bwanji?
  4. Mukanakhala kuti inu ndi amene mukukumana ndi mavuto amenewa kodi mukanalankhula ndi ndani za mavutowa?
  5. Mukanakhala kuti inu ndi amene mukukumana ndi mavuto amenewa kodi mukanathana nawo bwanji?
  6. Kodi mukuganiza kuti zina mwa zifukwa zimene mayiyu akukumana nazo zimene zikuyambitsa kukhumudwa kapena nkhawa (depression)?
  7. Pakanakhala chiyeso chimene chikanapeza munthu chonchi wokhala ndi vuto la kukhumudwa)” kodi mukuganiza kuti angamve bwanji zokhudza zimenezi?(Yesetsani kufufuza mmene zimakhalira kukhala ndi chiphinjo cha zones ziwiri; HIV ndi matenda okhumudwa.

5.Tonse tili ndi mavuto ngati amenewa nthawi zina pa nthawi zosiyanasiyana mu miyoyo yathu, Nthawi yyoyembekezela ndi nthawi yofunika mu moyo wa mzimayi. Mungandiuze mmene mwakhala mukumvela komanso zomwe zakhala zikuchitika mu moyo wanu kuchokela nthawi imene munadziwa kuti ndinu oyembekezela kapena nthawi imene munachira?

a. Ndizobvuta ziti zomwe mukukumana nazo? Kupezeka ndi HIV kwakhudza bwanji moyo wanu?

- 1. Kodi zinatenga nthawi yayitali bwanji?
  2. Kodi munalankhula ndi ndani za izo?
  3. Kodi munachita chiyani zokhudza zimenezi?
  4. Kodi izi zinakhudza bwanji chifuniro chanu cholandira mankhwala anu a HIV ndikupita ku maulendo anu a chisamaliro cha HIV?

**Chithandizo cha kukhumudwa**:

6. Ndi njira ziti, ngati zilipo, kodi mukuganiza kuti ndi zothandiza kwambiri kwa wina amene akukumana ndi mavuto amenewa?

1. Nanga ngati panali wina wolankhula naye ku kiliniki (kuganizira za uphungu woperekedwa ku kiliniki)?
2. Kodi mukuwona kuti zingakhale zothandiza bwanji'kwa munthu amene akukumana ndi mavuto amenewa pokambirana ndi wina ku kiliniki?
3. Kodi mukuganiza zingakhale bwanji kuti mukambirane ndi munthu ku kiliniki panokha kapena kukhala ndi amayi ena amene ali ndi mavuto ngati omwewa?
4. Ndi ndani kapena njira zina zingakhale ziti za wina amene akukumana ndi mavuto amenewa kuti alandire chithandizo?
5. Kodi mungaganize chiyani patakhala kuti pali mankhwala amene angawathandize, chimodzimodzi mmene odwala malungo atha kulandira mankhwala kuti iwo apeze bwino? (Note: Anthu odwala matenda okhumudwa amamwa pilisi limodzi kufikila atapeza bwino)
6. Kodi mukuganiza kuti anthu ambiri amamva bwanji zokhudza kumwa mankhwala pa nkhani ya thanzi?
   - 1. Kodi mukuganiza kuti anthu ambiri angamve bwanji zokhudza kumwa mankhwala a vuto lokhudza ku ubongo kapena kukhumudwa? (Nthawi yoyembekezela kapena yoyamwitsa)

7. Kwa amayi amene ali woyembekezera, kodi mukuganiza kuti kupezeka ndi HIV pa nthawi imene ali woyembekezera kutha kusokoneza ngati angathe kukhala ndi vuto la kukhumudwa (depression) kapena ayi?

8. Ngati mayi akukumana ndi vuto la kukhumudwa (depression) pa nthawi yoyembekezera, kodi mukuganiza kutizingasokoneze bwanji machitachita ake mchisamaliro cha HIV?

1. Kodi kukhumudwa (depression) kungasokoneze bwanji chifuniro cha amayi kumwa mankhwala awo a HIV? Nanga pa nkhani yopita ku maulendo awo a HIV?
