# Supplementary material for: "Pain in my heart": Understanding perinatal depression among women living with HIV in Malawi
Source: PLoS One. 2020 Jun 5;15(6):e0227935. doi: 10.1371/journal.pone.0227935 (PMC7274419; doi:10.1371/journal.pone.0227935)
Supplement: S2 Data — (DOCX) [file pone.0227935.s002.docx]

**Perinatal Depression and HIV – Women’s Interview Guide**

**Overview (for interviewer)**: The goal of this in-depth interview guide is to explore HIV-infected women’s experiences of perinatal depression (PND) and to gather information about their preferences for PND screening and treatment and how PND has affected their engagement in HIV care. In-depth interviews will include both prenatal and postnatal HIV-infected women with PND.

-------------------------------------------------------------------------------------------------

**Introduction:**

Hello, I am working on behalf of researchers at UNC Project-Malawi. We are very interested in hearing your opinion on some of the problems in this community, particularly problems that have to do with peoples’ behaviors during the time they are pregnant or right after they have a baby.

**Overview of Perinatal Depression:**

1. Have you ever noticed someone who has trouble with things when they are pregnant or soon after the baby comes that most people do not? For instance, trouble taking food, trouble engaging with the baby, or friends and family in a normal way (perhaps someone who isolates themselves)? Trouble doing household calls or even abusing substances to help cope with their problems? Possibly someone who is even thinking about harming themselves?
   1. How would you describe someone like this?

If no answer, further probe: What do you call it when someone is unhappy almost all of the time? What do you call it when this happens around the time of pregnancy?

1. Everyone feels sad, stressed, or worried from time to time. However, if the sadness or worries last a long time or are very severe, we would describe that as depression (matendu kukhumwudwa). Have you heard those words before?
   1. If so, how have you heard them used?
   2. If not, how would you describe when women experience sadness or difficulty coping during pregnancy?

**Depression Scenarios**:

1. Imagine a scenario involving a young mother with kukhumudwa (depression). Picture her being unhappy from time to time, perhaps crying often or being very quiet or even isolating herself from others when she is pregnant. Imagine after the baby is born, her having trouble motivating herself to care for the baby, managing household calls, or visit with friends and relatives when they come to meet the baby.
   1. How is someone like this treated by the community?
   2. How is someone like this treated by her family and friends?
   3. How is someone like this treated by her husband?
   4. If you were someone experiencing these problems who would you talk to about it?
   5. If you were someone experiencing these problems how would you deal with it?
   6. What do you think some of the reasons are that this woman might be experiencing worries or concerns during or after pregnancy?
   7. If there were a test that diagnosed a person like this as “being depressed (or having kukhumudwa)” how do you think they would feel about it?

Note: *If women talk about HIV above, take this opportunity to probe further on that distinction or you can skip this scenario.*

1. Now imagine a scenario where during pre-natal care, or around the time the baby is born, the mother finds out that she has been diagnosed with HIV. Picture her being unhappy, perhaps crying often. She may be having trouble motivating herself to care for the baby, managing household calls, or visit with friends and relatives when they come to meet the baby.
   1. How is someone like this treated by the community?
   2. How is someone like this treated by her family and friends?
   3. How is someone like this treated by her husband?
   4. If you were experiencing these problems who would you talk to about it?
   5. If you were experiencing these problems how would you deal with it?
   6. What do you think some of the reasons are that this woman might be experiencing such worries or anxiety during pregnancy or after the baby is born?
   7. If there were a test that diagnosed a person like this as “being depressed (or having kukhumudwa)” how do you think they would feel about it (*try to get at what it is like having the dual burden of HIV and depression)*?
2. We all have difficulties like these to some degree at different times in our lives., Pregnancy and having a baby is an important time in a woman’s life. Can you tell me about how you’ve been feeling or what’s been going on in your life since you found out you were pregnant or had your baby?
   1. What challenges are you experiencing? How has your HIV diagnosis affected you?
   2. How long did it last for?
   3. Who did you talk to about it?
   4. What did you do about it?
   5. How did it affect your ability to take your HIV medications and go to HIV appointments?

**Depression Treatments**:

1. What ways, if any, do you think are most helpful to someone who is experiencing these problems?
   1. What if there was someone to talk to at the clinic (gauging thoughts about counseling from a clinic)?
   2. How effective do you think it might be for someone who is experiencing these problems to discuss them with someone at a clinic?
   3. What do you think about talking with someone at the clinic by yourself or with other women experiencing similar issues?
   4. Who or what might be some other ways for someone experiencing these problems to seek help?
   5. What would you think if there were a medication that could help them, the same way someone with Malaria can take a medication to make them better? [note: *people with depression often take 1 pill per day until they start to feel better*.]
   6. How do you think most people feel about taking medications for health issues?
      1. How do you think most people would feel about taking medication for mental health issues or kukhumudwa during pregnancy and breastfeeding?
2. For women who are pregnant, how do you think being diagnosed with HIV during pregnancy affects whether or not they develop kukhumudwa (depression)?
3. If a woman experiences kukhumudwa (depression) during pregnancy, how do you think it may affect her engagement in her HIV care?
   1. How might kukhumudwa (depression) affected women’s ability to take their HIV medications? What about attending their HIV appointments? During pregnancy? What about after the baby is born?
