# Supplementary material for: "Pain in my heart": Understanding perinatal depression among women living with HIV in Malawi
Source: PLoS One. 2020 Jun 5;15(6):e0227935. doi: 10.1371/journal.pone.0227935 (PMC7274419; doi:10.1371/journal.pone.0227935)
Supplement: Attachment — Submitted filename: PONE-D-20-00046.docx [file pone.0227935.s003.docx]

**Dear Editor in Chief, Dr. Montazeri**

PONE-D-20-00046

"Pain in my heart": Understanding perinatal depression among women living with HIV in Malawi.

The comments for the above manuscript:

**Short Title:**

Short title is just like the main title so it must be corrected.

**Abstract:**

- In the Methods: The research method is not mentioned.

**Introduction:**

- The beginning sentences for the introduction seems to be not suitable because the HIV treatment is not the main problem of the present study.
- Line 45-48: It is not clear, what does it mean?
- The authors have noted that the disease is associated with depression, so the need for a study is unclear. They have mentioned to “women living with HIV in low income settings” but there is not any explanation about the reasons for probable differences of the PND in this group of women with others that previous studies have conducted on them. So the necessity of the present study should be clarified.

**Methods:**

- How many women completed the EPDS questionnaire? What was the sampling method?
- EPDS questionnaire is a postnatal depression scale. How the authors explain for using it during pregnancy?
- What was the evidence for living with HIV?

**Results:**

- Is written in a pleasant and expressive way.
- Table 1: It is better to write the percentages in the last two columns next to their related numbers (4^th^ and 5^th^ column) in the parenthesis.

**Discussion:**

- Is well written.

With best regards

Hedyeh Riazi
