# Supplementary material for: "Pain in my heart": Understanding perinatal depression among women living with HIV in Malawi
Source: PLoS One. 2020 Jun 5;15(6):e0227935. doi: 10.1371/journal.pone.0227935 (PMC7274419; doi:10.1371/journal.pone.0227935)
Supplement: Attachment — Submitted filename: LeMasters_ResponseReviewers_04.06.20.docx [file pone.0227935.s004.docx]

Response to Reviewers

We very much appreciate the time taken to review our manuscript and for your thoughtful comments.

**Reviewer #1:**

**Short Title:**

Short title is just like the main title so it must be corrected.

Thank you for this, it is now “Perinatal depression and HIV in Malawian Women.”

**Abstract:**

- In the Methods: The research method is not mentioned.
  - Thank you for this. We have only included a brief description of our methods in the abstract due to word limitations, but we have been sure to include our sample size and that we used both inductive and deductive coding and narrative analysis (**page 2 lines 27-29**). I have also added ‘qualitative’ in front of ‘interviews’ for further clarification.

**Introduction:**

- The beginning sentences for the introduction seems to be not suitable because the HIV treatment is not the main problem of the present study.
  - Thank you for your comment. While it is true that HIV treatment is not the only problem of this study, the statement is meant to provide a broad overview of how HIV treatment and maternal mental health intersect and may contribute to poor HIV outcomes. We hope that our revisions to what were lines 45-48 below help to clarify this.
- Line 45-48: It is not clear, what does it mean?
  - Thank you for your question. We have edited this statement, which is meant to connect poor HIV retention in care with maternal mental health (**page 2 line 47-50**), as our study explores the connection between the two.
- The authors have noted that the disease is associated with depression, so the need for a study is unclear. They have mentioned to “women living with HIV in low income settings” but there is not any explanation about the reasons for probable differences of the PND in this group of women with others that previous studies have conducted on them. So the necessity of the present study should be clarified.
  - Thank you for your comment. As you said, and as stated in the manuscript, we know that perinatal depression affects many women in low and middle-income countries and we know that adults living with HIV are at an increased risk for depression. However, very little is known about perinatal depression among women living with HIV. Indeed, what we do know is quantitative and/or is not specific to only women living with HIV (**added to page 3 line 77**).^1,2^ Thus, there is a need to develop a richer, qualitative understanding of PND specifically among HIV-positive women. Additionally, mental illness and its stigma is conceptualized differently in different settings, further indicating the need to deeply explore its intersection with HIV in various settings.^3^

**Methods:**

- How many women completed the EPDS questionnaire? What was the sampling method?
  - Under Results, Demographic Information (**page 6, line 145**), it states that 73 women were screened. This is also noted in the Abstract.
  - **Page 4, lines 93-95** states that women were sampled consecutively at each site until the desired sample size was reached. Consecutive sampling is very similar to convenience sampling. Each eligible patient who presents for care is approached for enrollment until the desired sample size has been reached.^4^ The citation has now been added to the manuscript.
- EPDS questionnaire is a postnatal depression scale. How the authors explain for using it during pregnancy?
  - Thank you for your question. While originally designed to screen for postpartum depression, the EPDS is frequently used to screen for depression in antenatal settings, including in sub-Saharan Africa, which is now included in the manuscript (**page 4, lines 91-92**).^5,6^
- What was the evidence for living with HIV?
  - Women’s health passports were used to determine their HIV status, this has been updated in the manuscript in the methods section (**page 4, lines 88-89**).

**Results:**

- Is written in a pleasant and expressive way.
  - Thank you for your feedback, it’s greatly appreciated.
- Table 1: It is better to write the percentages in the last two columns next to their related numbers (4^th^ and 5^th^ column) in the parenthesis.
  - Thank you for this suggestion, this has been done and Table 1 reflects these changes (**page 7**).

**Discussion:**

- Is well written.
  - Thank you so much for your comments.

**Reviewer #2:**

This article is technically and conceptually well organized. All the criteria of a research article is considered in writing.

Thank you very much for your review and comments.

I suggest some revision in line 85 as it is not clear whether 4 or 5 women were interviewed.
Also, I think it would be better to assign the statistical tests used in table 1 in lines 132-136.

Thank you for your comment. 24 women were recruited, requiring one site to have 4 women while the remaining sites recruited 5. This has been added to the manuscript (**page 4, lines 94-95)**. Our goal in qualitative interviews was to reach saturation, where new data are redundant with data already collected.^7^ Given the rule of thumb of 5-10 in-depth interviews per stratum (pre- and post-natal), we expected that we would achieve saturation within our proposed sample size. This has also been added to the manuscript (**page 4, lines 128-129**). We hope that this change clarifies the second part of your statement, as we are not sure what the statisticial tests are that you are referencing.

PLOS One Comments

1. Please ensure that your manuscript meets PLOS ONE's style requirements, including those for file naming. The PLOS ONE style templates can be found at <http://www.plosone.org/attachments/PLOSOne_formatting_sample_main_body.pdf> and<http://www.plosone.org/attachments/PLOSOne_formatting_sample_title_authors_affiliations.pdf>

2. Please include a copy of the interview guide used in the study, in both the original language and English, as Supporting Information, or include a citation if it has been published previously.

I am submitting a copy of the interview guide in both English and Chichewa.

3. Thank you for stating the following in the Acknowledgments Section of your manuscript:

"This research was supported by grant R34 MH 116806 and R00 MH 112413 from the National Institute of Mental health (NIMH) and by a developmental grant from the UNC Center for AIDS Research (CFAR), an NIH-funded program (P30 AI 050410). This paper does not reflect the views of the NIMH or NIH."

We note that you have provided funding information that is not currently declared in your Funding Statement. However, funding information should not appear in the Acknowledgments section or other areas of your manuscript. We will only publish funding information present in the Funding Statement section of the online submission form.

Please remove any funding-related text from the manuscript and let us know how you would like to update your Funding Statement. Currently, your Funding Statement reads as follows:

"The funders had no role in study design, data collection and analysis, decision to publish, or preparation of the manuscript."

Thank you for this, we would like the funding statement to be updated to be what was previously in the acknowledgements and has been updated to include the Fogarty International Center based on one of our co-authors current affiliations:

This research was supported by grant R34 MH 116806 and R00 MH 112413 from the National Institute of Mental health (NIMH) and by a developmental grant from the UNC Center for AIDS Research (CFAR), an NIH-funded program (P30 AI 050410). This work was also supported by the Fogarty International Center through the Malawi HIV implementation research scientist training program (D43- D43TW010060). This paper does not reflect the views of the NIMH, NIH, or Fogarty.

The acknowledgements have been updated accordingly (**page 19, lines 449-450**).

4. We note that you have indicated that data from this study are available upon request. PLOS only allows data to be available upon request if there are legal or ethical restrictions on sharing data publicly. For information on unacceptable data access restrictions, please see <http://journals.plos.org/plosone/s/data-availability#loc-unacceptable-data-access-restrictions>.

In your revised cover letter, please address the following prompts:

a) If there are ethical or legal restrictions on sharing a de-identified data set, please explain them in detail (e.g., data contain potentially identifying or sensitive patient information) and who has imposed them (e.g., an ethics committee). Please also provide contact information for a data access committee, ethics committee, or other institutional body to which data requests may be sent.

b) If there are no restrictions, please upload the minimal anonymized data set necessary to replicate your study findings as either Supporting Information files or to a stable, public repository and provide us with the relevant URLs, DOIs, or accession numbers. Please see <http://www.bmj.com/content/340/bmj.c181.long> for guidelines on how to de-identify and prepare clinical data for publication. For a list of acceptable repositories, please see <http://journals.plos.org/plosone/s/data-availability#loc-recommended-repositories>.

We will update your Data Availability statement on your behalf to reflect the information you provide.

These data also contain sensitive patient information, as all women are HIV-positive and suffering from perinatal depression. Furthermore, in our informed consent forms, it says "We will not share the information you give us with anyone not involved in the study." The ethics committee that imposes this the National Health Sciences Research Committee in Malawi, as we used their informed consent forms. The committee can be reached at: Tel: +265 1 726 422/418 or Email: [mohdoccentre@nhsrc-mw.com](mailto:mohdoccentre@nhsrc-mw.com).

5. Your ethics statement must appear in the Methods section of your manuscript. If your ethics statement is written in any section besides the Methods, please move it to the Methods section and delete it from any other section. Please also ensure that your ethics statement is included in your manuscript, as the ethics section of your online submission will not be published alongside your manuscript.

Thank you for this, an ethics statement was located in the methods section but has now been updated to match exactly the ethics statement that was submitted separately (**page 6; lines 172-176**).

Bibliography

1.	Davies T, Schneider M, Nyatsanza M, Lund C. “The sun has set even though it is morning”: Experiences and explanations of perinatal depression in an urban township, Cape Town. *Transcult Psychiatry*. 2016;53(3):286-312.

2.	Dow A, Dube Q, Pence BW, Van Rie A. Postpartum Depression and HIV Infection among Women in Malawi. *J Acquir Immune Defic Syndr*. 2014;65(3):359-365. doi:10.1038/jid.2014.371

3.	Semrau M, Evans-Lacko S, Koschorke M, Ashenafi L, Thornicroft G. Stigma and discrimination related to mental illness in low- and middle-income countries. *Epidemiol Psychiatr Sci*. 2015;24(5):382-394. doi:http://dx.doi.org/10.1017/S2045796015000359

4.	Martínez-Mesa J, González-Chica DA, Duquia RP, Bonamigo RR, Bastos JL. Sampling: how to select participants in my research study? *An Bras Dermatol*. 2016;91(3):326-330. doi:10.1590/abd1806-4841.20165254

5.	Hung KJ, Tomlinson M, le Roux IM, Dewing S, Chopra M, Tsai AC. Community-based prenatal screening for postpartum depression in a South African township. *Int J Gynecol Obstet*. 2014;126(1):74-77. doi:10.1016/j.ijgo.2014.01.011

6.	Malqvist M, Clarke K, Matsebula T, Bergman M, Tomlinson M. Screening for antepartum depression through community health outreach in Swaziland. *J Community Health*. 2016;41(5):946-952. doi:10.1038/leu.2016.282.Dynamic

7.	Glaser B, Strauss A. *The Discovery of Grounded Theory: Strategies for Qualitative Research.* Chicago: Aldine Publishing Company; 1967.
